# A cooperative deep learning model for stock market prediction using deep autoencoder and sentiment analysis

**DOI:** 10.7717/peerj-cs.1158

**Published:** 2022-11-30

**Authors:** KS Rekha, MK Sabu

**Affiliations:** 1 Department of Computer Applications, Cochin University of Science and Technology, Kochi, Kerala, India; 2 Department of Computer Science and Engineering, College of Engineering Kidangoor, Kottayam, Kerala, India

**Keywords:** Stock market prediction, Deep learning, Sentiment analysis

## Abstract

Stock market prediction is a challenging and complex problem that has received the attention of researchers due to the high returns resulting from an improved prediction. Even though machine learning models are popular in this domain dynamic and the volatile nature of the stock markets limits the accuracy of stock prediction. Studies show that incorporating news sentiment in stock market predictions enhances performance compared to models using stock features alone. There is a need to develop an architecture that facilitates noise removal from stock data, captures market sentiments, and ensures prediction to a reasonable degree of accuracy. The proposed cooperative deep-learning architecture comprises a deep autoencoder, lexicon-based software for sentiment analysis of news headlines, and LSTM/GRU layers for prediction. The autoencoder is used to denoise the historical stock data, and the denoised data is transferred into the deep learning model along with news sentiments. The stock data is concatenated with the sentiment score and is fed to the LSTM/GRU model for output prediction. The model’s performance is evaluated using the standard measures used in the literature. The results show that the combined model using deep autoencoder with news sentiments performs better than the standalone LSTM/GRU models. The performance of our model also compares favorably with state-of-the-art models in the literature.

## Introduction

Stock markets offer an opportunity to invest and gain from the growth of an industry or organization. Investment in the stock is perceived as risky, considering the various factors influencing the fluctuations of the stock prices and the instability of stock markets. Stock markets are complex, volatile, and chaotic, and stock price prediction is a non-trivial problem. The need for improved prediction in this domain is recognized, and researchers have been trying to evolve models that support the prediction of stock prices.

Accurate stock price prediction is a challenging problem due to the dynamic and volatile nature of the stock market. Stock markets are subject to fluctuations due to different causes, and determining the movement of stock price helps minimize investors’ risk (Mohanty, Parida & Khuntia, 2021).

Due to stock data’s dynamic, nonlinear, and non-parametric nature (Li & Bastos, 2020), statistical models were initially explored in stock market prediction (Kumar, Sarangi & Verma, 2021), but their applications were limited by weak performance of the models. Researchers started to explore the machine learning and deep learning techniques in stock prediction (Deepak, Uday & Malathi, 2017) to improve performance compared to that of statistical models. Machine learning algorithms, to a great extent, can give satisfactory results on stock market prediction (Parmar et al., 2018).

Strader et al. (2020) described the research direction of the stock market prediction domain, specifically using Artificial Neural Networks (ANN), meta-heuristics, Support Vector Machines (SVM), and Artificial Intelligence (AI) based techniques. We can find a non-linear relationship in a dataset without having apriori information of dependency of input and output in an ANN (Kurani et al., 2021) which is an advantage of ANN. In regression problems involving complex functions, due to overfitting and getting trapped in local minima, the ANNs cannot generally provide accurate results.

Researchers focused on using the Multilayer Perceptron in stock market prediction (Pan, Tilakaratne & Yearwood, 2005; Devadoss & Ligori, 2013) decades ago. Later, different ANN models and hybrid algorithms were used. Stock market data is noisy, and ANN exhibits unpredictable and inconsistent behavior on this data (Guresen, Kayakutlu & Daim, 2011). Deep learning techniques are found to be helpful in stock performance prediction (Singh & Srivastava, 2017). Some of the drawbacks of ANN can be overcome by using Deep Neural Networks (DNN) as they give a better approximation to nonlinear models (Yu & Yan, 2020; Nabipour et al., 2020). DNNs give better performance when applied to some time series forecasting (Le Roux & Bengio, 2010; Sutskever & Hinton, 2008). Takeuchi & Lee (2013) proposed a study that uses autoencoders and restricted Boltzmann machines for feature extraction from the input variables for stock trading strategies.

Machine learning techniques enable the prediction of stock price by integrating technical indicators, financial news and social media posts (Albahli et al., 2022). Stock price prediction problem can be modeled as a time series forecasting problem involving past data and other attributes like news sentiments (Bhardwaj et al., 2015; Mehta, Pandya & Kotecha, 2021). Some studies use sentiment analysis methods, stock price datasets, and some works integrating stock price datasets and sentiment analysis are discussed in the literature review in Section 2. With the rapid developments in soft computing and different computational techniques in the last decades, researchers have proposed many methods based on artificial neural networks, heuristics, and meta-heuristics for stock market prediction (Araújo, 2010; Vui et al., 2013; Shi & Zhuang, 2019; Lv et al., 2019; Atsalakis & Valavanis, 2009; Haider Bangyal et al., 2022; Pervaiz et al., 2021). News has a significant impact on stock prices. News articles are available publicly online, and we can extract valuable data and information from them Khan et al. (2019). The type of news data useful in stock market prediction can be categorized as general news, financial news, and political news.

Despite the dynamic growth of the real stock market, integrating news sentiment, historical stock data, and autoencoders is an exciting research domain. We propose a method incorporating deep learning models and a deep autoencoder combined with sentiment analysis for improved stock prediction. The integrated architecture involving these three aspects facilitates an efficient prediction as the autoencoder minimizes the effect of noise in the data to complement the prediction capability of the deep learning model.

The remainder of the paper is structured as follows. Section 2 describes a detailed literature review on stock market prediction techniques and methods. Section 3 provides the article contribution of the research, and Section 4 gives methodology that includes the dataset, features, and proposed architecture. This section describes the working of the model using a schematic diagram. Besides, different aspects of deep learning used in modeling are also described. Section 5 presents the results and analysis obtained from this study, followed by discussions. The paper concludes with Section 6 which also includes future work.

## Related Work

We discuss published literature in three different categories here. These categories are related to using deep learning models in stock prediction, autoencoders in financial time series prediction, and sentiment analysis, respectively. The state-of-the-art models pertaining to these domains is reviewed in detail and elaborated.

### Deep learning techniques in stock prediction

Deep learning, an advanced version of machine learning, has an excellent capability for information extraction from time-series data (Nabipour et al., 2020). Since stock price prediction is a sequence prediction time series problem, one of the most suited architectures for this is Recurrent Neural Networks (RNN). RNNs and variants of RNNs perform well on stock market prediction. Roondiwala, Patel & Varma (2017) have done a study using RNN and LSTM. Nelson, Pereira & de Oliveira (2017) also predicted whether a stock would move up or down using LSTM.

LSTM algorithms are suitable for prediction involving non-linear data (Rajakumari, Kalyan & Bhaskar, 2020). Chen, Zhou & Dai (2015) applied an LSTM model for stock price prediction problems concerned with the Chinese stock market. This model comprises of an input, LSTM, and an output layer with multiple neurons. This work showed that the normalized features could increase the forecast accuracy. In this work, they have not considered the news sentiments. Shahi et al. (2020) have done a study incorporating the sentiments using the news headlines. A comparison was made to evaluate the relative performance of LSTM and GRU models in Rahman et al. (2019).

A deep neural network with connections between the layers, composed of multiple hidden units but not between units within layers, is termed a deep-belief network. In work done by Wang et al. (2018), the backpropagation neural network model and the LSTM model are used to find the prediction accuracy. Pawar, Jalem & Tiwari (2019) used RNN along with LSTM for stock market prediction. They have compared the performance of the model with the common algorithms like random forest, feed forward neural network, Support Vector Machine, and backpropagation. Deep learning methods are useful in stock price prediction and in other domains of finance like portfolio management (Soleymani & Paquet, 2020). Increasing the expected return on investment by proper rationing of the investment amount into the different components of the portfolio is the objective of portfolio management.

### Autoencoder

Bao, Yue & Rao (2017) used stacked autoencoders and LSTM for the financial time series prediction problem. In this study, the input financial time series data is passed through a multi-resolution discrete wavelet transformation, and the denoised financial time series is passed through a stacked autoencoder to get the one-step-ahead output using LSTM. Song et al. (2022) have shown that deep autoencoders are useful in prediction tasks. Gunduz (2021) obtained results with reasonable accuracy for the Istanbul stock market by performing stock market prediction using autoencoder and LSTM with attention.

The architecture of a deep autoencoder comprises two deep belief networks with more than three shallow layers for encoding and the next layer set for the decoding half. One highlight of our paper is that it uses a supervised model as well as an unsupervised model. Due to the dynamic nature of stock markets, even predicting the direction of the stock movement is a challenging task (Li et al., 2017). They have used a system of stacked denoising autoencoders to predict the movement of stock indexes. Deep learning architecture including autoencoders is efficient in complex learning problems with insufficient samples and uncertain information as demonstrated by Zheng et al. (2021).

Denoising of data is a crucial aspect of stock price prediction. To denoise data, Liu et al. (2019) used autoencoders, and LSTM is used for prediction. In short-term electric load forecasting, autoencoders have been successfully used by Liu, Zheng & Chen (2019). The analysis was done with data on Chinese cities and found that the prediction error decreases using autoencoders compared to the traditional backpropagation (BP) neural networks. Although LSTM-based methods provide better performance than conventional methods in time series prediction, it has inherent limitations in modeling multivariate data. LSTM and stacked autoencoders were used by Sagheer & Kotb (2019) to validate the model’s performance.

### Sentiment analysis

Sentiment analysis involves the detailed analysis of text with opinions and emotions. Sentiment analysis seeks to find whether the subjective parts of the text contain positive or negative sentiments. Studies show that sentiments positively or negatively impact stock market forecasting. In this research, we are considering the news headlines. The positive news is assigned a value of 1, and the negative information is assigned a value of 0.

The use of deep learning models along with sentiment analysis is considered a relevant research domain due to the dynamic nature of the stock markets (Bhardwaj et al., 2015; Yadav et al., 2019; Jiawei & Murata, 2019; Moghar & Hamiche, 2020; Shahi et al., 2020; Mehta, Pandya & Kotecha, 2021). Mehta & Pandya (2020) presents a comprehensive review of machine learning approaches to identify sentiments from a context.

Bhardwaj et al. (2015) used news sentiments and historical stock prices to predict the stock market. In this work, news articles and stock values are preprocessed, the sentiments are classified, and the analysis is performed using the random forest method. Thus prediction is made, and visualization of stock data is performed. Carosia, Coelho & da Silva (2021) proposes strategies for investment based on sentiment analysis in the context of the Brazilian stock market. Nemes & Kiss (2021) performed stock movement prediction using stock news headlines using different sentiment analysis tools. Stock prediction incorporating investor sentiments and stock data is effective, as demonstrated by Wu et al. (2022) based on Chinese stock market data.

To predict the stock market, Yadav et al. (2019) used a combination of machine learning algorithms and lexicon-based labeling of financial news. The ‘market emotion’ is a significant factor influencing the stock market forecasts, as indicated in Jiawei & Murata (2019), and in their work, they used LSTM to predict stock trend prediction. Feature selection was used to select useful indices of stock and use a deep learning model to do financial news sentiment analysis to predict stock trends. Deorukhkar et al. (2019) used LSTM networks, ARIMA, and Sentiment Analysis to predict the stock price. Natural language processing methods are used to bridge the stock market indices and the news sentiments to improve the performance of stock market prediction (Seng & Yang, 2017).

In the problems involving sentiments, the lexicon-based approach is robust and provides reasonably good results (Hailong, Wenyan & Bo, 2014). We have adopted Valence Aware Dictionary and sEntiment Reasoner (VADER), a lexicon and rule-based sentiment analysis tool, in our model. Each word is linked with a sentiment polarity value, and a predefined dictionary of words is used in a dictionary-based method (Bhonde et al., 2015).

Business sentiment can be effectively captured from news text based on newspaper articles (Seki, Ikuta & Matsubayashi, 2022). Mehta, Pandya & Kotecha (2021) have proposed models for stock price prediction by integrating sentiment analysis and different machine learning techniques. In addition, the association between news sentiments and stock trends was also explored. Gite et al. (2021) have also shown that prediction accuracy can be enhanced by fusing sentiments along with technical analysis. Prediction of stock market movement during the COVID-19 pandemic was made by Das et al. (2022) using different sentiment analysis tools and LSTM. As evident from the above works, using market sentiments captured from news articles can improve stock price prediction accuracy.

## Article Contribution

To the best of our knowledge, research incorporating news sentiment analysis, historical stock price data, and deep autoencoders for noise reduction in a single stock prediction system is not available in the literature. Since time series problems such as stock market prediction are sequence prediction problems, it is crucial to consider the sequential relationships among the data. To address these research gaps, we have tried to:

1. Develop an integrated architecture based on deep learning with features captured from previous stock price data and a deep autoencoder for noise removal.
2. Understand the impact of news sentiment on stock market prediction.
3. Find which model of deep-learning, GRU or LSTM, performs better in stock market prediction.

High stakes and incentives for better stock prediction demand more accurate models that can improve the prediction accuracy.

## Methodology

In this section, we first introduce the proposed architecture used in our model. Later we discuss the deep learning components of the model, deep autoencoder, LSTM, and GRU, and the preprocessing methods used in this study. We name the models DeepAutoEncoder-Long Short Term Memory Sentiment Analysis (DAE-LSTMSA) and DeepAutoEncoder-Gated Recurrent Unit Sentiment Analysis (DAE-GRUSA).

### Proposed architecture

**Figure 1:**
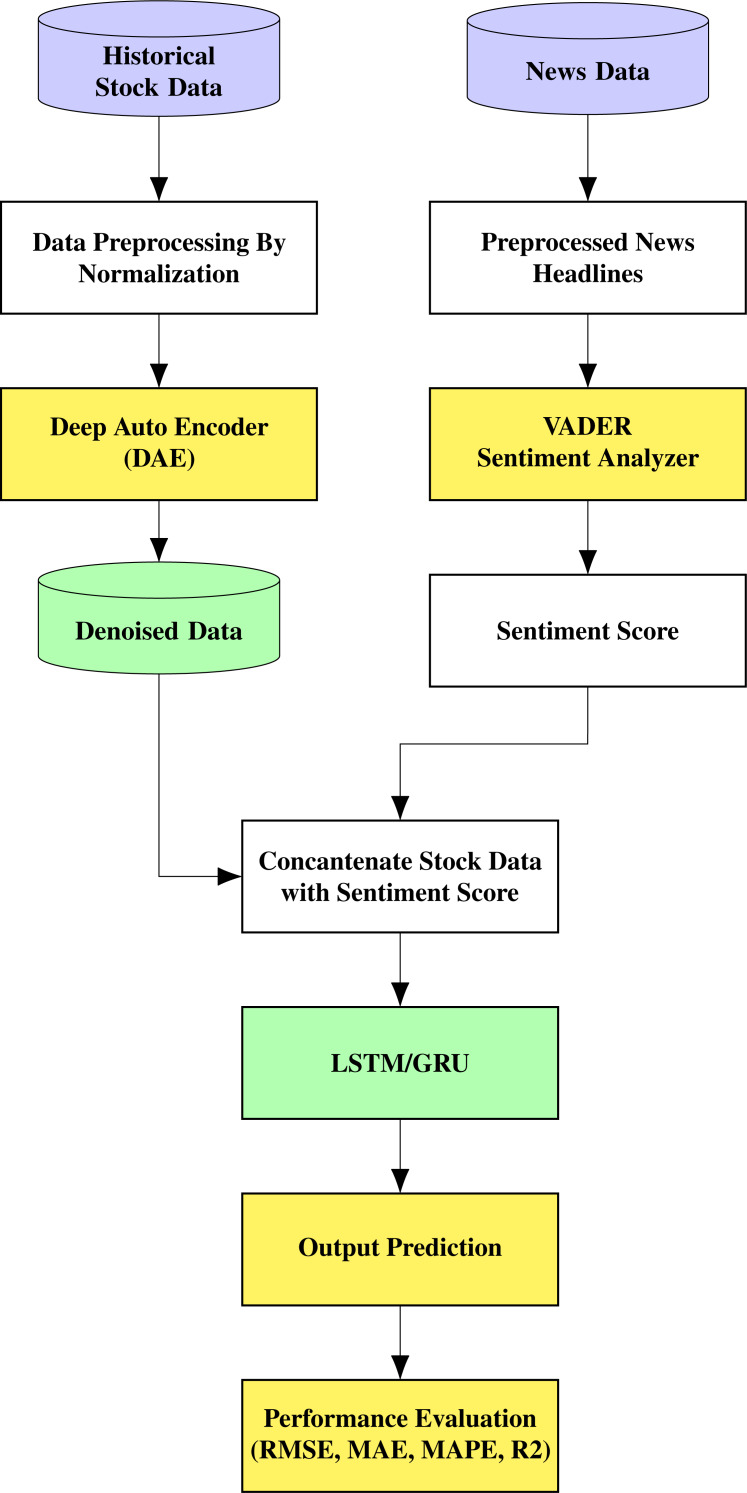
Proposed architecture.

The proposed architecture is shown in Fig. 1. The stock prediction is made through a multi-stage approach comprising denoising the data using an autoencoder, which is fed to an LSTM/GRU model that also receives information based on sentiment analysis. The noisy historical stock market data is fed into the autoencoder, where it undergoes encoding and decoding. The autoencoder used in this work is unsupervised learning or self-supervised learning since it does not require any human intervention, such as data labelling.

The encoder transforms the input into a lower-dimensional representation, and the decoder tries to reconstruct the original data from the lower-dimensional representation. Therefore, the objective is to restore the initial data with the minimum amount of information loss. Stock prices for each day and news related to the company for the same period are contained in this data. The top 25 news on a day related to the stock is taken into consideration for the study. The daily stock prices include six values termed Open, High, Low, Close, Adjusted Close, and Volume. We are considering the Open, High, Low, and Close (OHLC) values in this research collected from Yahoo finance.

The news dataset used in this study for sentiment analysis with news headlines is extracted from the Kaggle community. The top 25 news from the period 01/03/2000 to 01/07/2016 was used in this study. After separating and removing the unwanted features, the top 25 news was concatenated into a single string for a single day. This process was continued for all days. After the news string of a single day was obtained, it was then combined with the appropriate date (time series) and the stock market data of the particular company. This process is called the pickling of the file, in which we combine the news articles in string format along with stock data in integer format. In the sentiment analysis part with the news dataset, we collated the headlines of the articles and dates. Later, by extracting and cleaning the data, we performed text processing.

### Preprocessing

Data preprocessing means converting raw data into efficient and useful data. The stock data is noisy, and the noise is to be removed. To denoise, the data we pass through a deep autoencoder, and the output we obtain is a denoised historical stock dataset. We have done min-max data normalization using Eq. (1) to make all values within the range of 0 and 1. This improves the accuracy, increases the gradient descent rate, and generates the optimal solution (Ding & Qin, 2020).

(1) \begin{eqnarray*}y= \frac{x-{x}_{min}}{{x}_{max}-{x}_{min}} .\end{eqnarray*}

In Eq. (1) the minimum (*x*_*min*_) and maximum values (*x*_*max*_) relate to the value x being normalized. The normalized data is fed into the autoencoder for further processing. Data normalization aims to ensure quality data to the central parts of the model.

The text data is unstructured, and hence preprocessing is necessary before feeding it to the model. We create a list of the news section URL of the component companies and then extract the relevant news article’s web links from the company’s news section page. We remove the duplicate news articles based on news titles, and the main text from the selected news articles is extracted. By concatenating all the article headlines, a single string was formed for a single day after processing the relevant articles. After obtaining the single string for a day, it is merged with the appropriate date and stock index value. We compute the sentiment analysis score generated from textual data, which is added to the stock data and is used as the input of the prediction model.

### Deep autoencoder

The noisy stock market data is fed into the DAE, which undergoes encoding and decoding. The DAE used in this work is unsupervised learning or self-supervised learning since it does not require human intervention such as data labeling (Skansi, 2018). The encoder transforms the input to a lower dimensional representation, and a decoder reconstructs the original data from the lower dimensional representation. Therefore the objective is to restore the initial data with the minimum amount of information loss. A DAE with two symmetrical deep belief networks having an encoding half and the second with a decoding half is used here. In the encoder part, the input data is compressed to reduce the relevant information, which results in significant size reduction. The decoder produces the reverse operation of the encoder, *i.e.,* the uncompressed data is created as a reconstruction of the input as accurately as possible.

**Figure 2:**
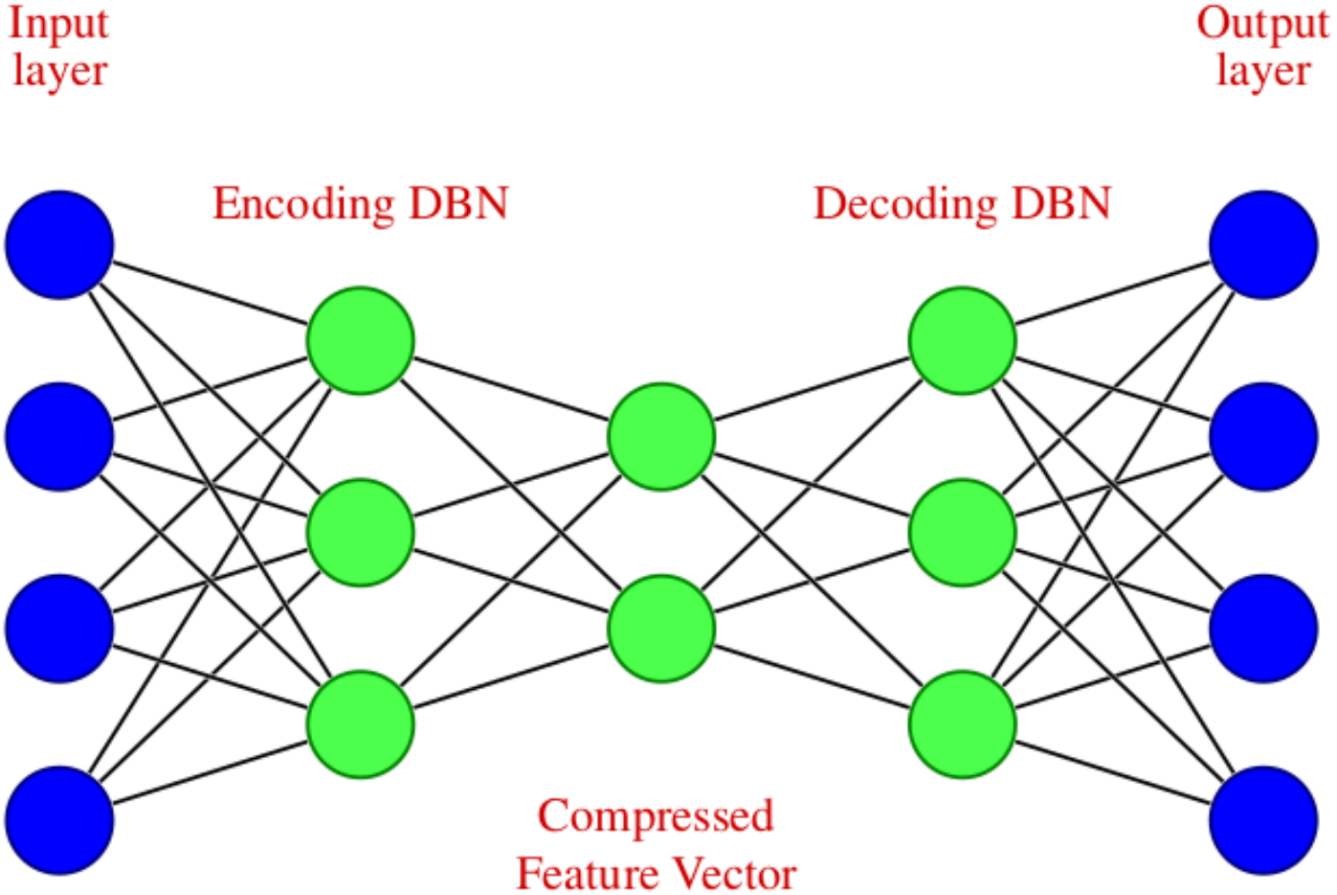
Deep Autoencoder with four input and output features.

The DAE we have used in modeling can be diagrammatically represented as shown in Fig. 2, which consists of an input layer, encoding deep belief network, compressed feature vector, decoding deep belief network, and an output layer.

The DAE structure is shown in Table 1 which consists of different layers such as input and dense layer, the shape of the output, and different parameters.

The notations used in the DAE are given in Table 2. The process of encoding and decoding in the DAE follows Eqs. (2) and (3). The DAE is trained by error minimization as shown in Eq. (4).

**Table 1:** Deep autoencoder structure.

Layer (Type)	Output shape	Param #
input_1 (InputLayer)	(None, 4)	0
dense_1 (Dense)	(None, 80)	400
dense_2 (Dense)	(None, 50)	4050
dense_3 (Dense)	(None, 80)	4080
dense_4 (Dense)	(None, 4)	324

**Table 2:** Deep autoencoder notations and descriptions.

**Notation**	**Description**
*S*	Input data to encoder, *S* = {*s*_1_, *s*_2_, …, *s*_*n*_}
*T*	Original data characteristic sequence, *T* = {*t*_1_, *t*_2_, …, *t*_*n*_}
*D*	Output data from decoder *D* = {*d*_1_, *d*_2_, …, *d*_*n*_}
*f*, *g*	Sigmoid functions
*w*	Weights
*b*	Biases

(2) \begin{eqnarray*}{t}_{i}=f({w}_{t}.{s}_{i}+{b}_{t})\end{eqnarray*}

(3) \begin{eqnarray*}{d}_{i}=g({w}_{d}.{t}_{i}+{b}_{d})\end{eqnarray*}

(4) \begin{eqnarray*}E(S,D)= \frac{1}{2} \sum _{i=1}^{n}{|}{|}{s}_{i}-{d}_{i}{|}{{|}}^{2}\end{eqnarray*}

### LSTM model

**Figure 3:**
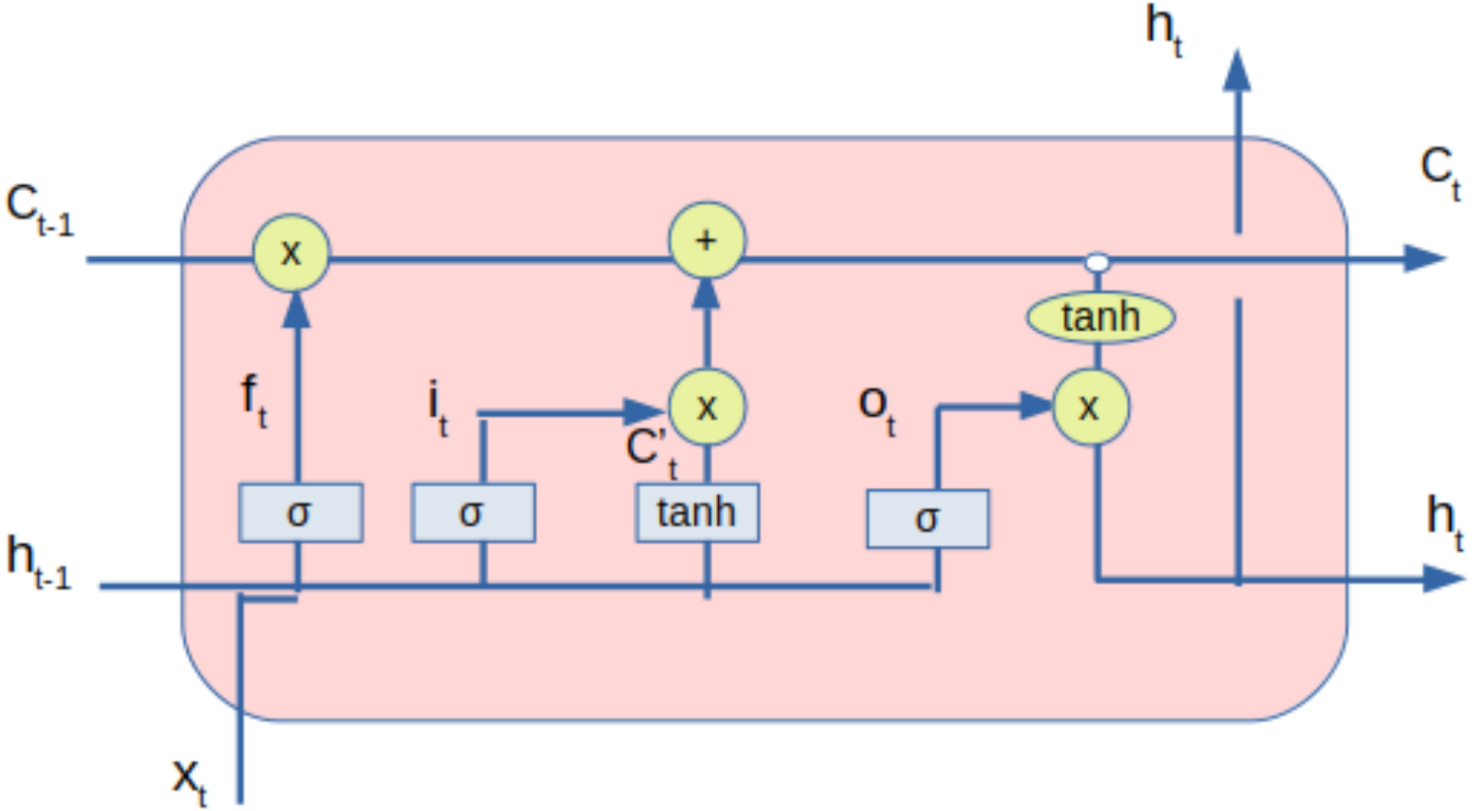
LSTM with inputs and outputs.

The processed data from the DAE, along with sentiment value, is provided to the LSTM network. LSTM is used as it can capture long-term dependencies, thereby eliminating the problem of vanishing gradient (Hochreiter, 1998). LSTM helps in feature extraction from data using its multi-layered architecture. The number of neurons in different layers and the number of layers impact the performance. The LSTM can be diagrammatically represented as shown in Fig. 3.

Table 3 represents the notations and descriptions used in LSTM.

The Eqs. (5), (6) and (7) represents the forget gate, input gate and output gate respectively. The candidate state and state of the memory cells at time *t* are given by the Eqs. (8) and (9) respectively whereas the Eq. (10) represents the equation for the hidden state.

**Table 3:** LSTM notations and descriptions.

**Notation**	**Description**
*f*_*t*_	Forget gate
*i*_*t*_	Input gate
*o*_*t*_	Output gate
*C*_*t*_	Cell state
*h*_*t*_	Hidden state
*σ*	sigmoid
tanh	tanh
*W*, *U*	Weights
*b*	Bias
⊙	Element wise multiplication

The inputs provided to the LSTM are cell states and hidden states of time-step *t* − 1. The outputs of the LSTM are cell states and hidden states of time-step t. Forget gate dictates the extent of information that can be input into the LSTM unit. The input gate controls the extent of information that can be learned. Tanh is the activation layer that maps inputs into cell states. Output gate controls the information that can be output as a hidden state for the next time-step. Tanh activation layer activates the information of the cell state.

(5) \begin{eqnarray*}{f}_{t}=\sigma ({W}_{f}\times {x}_{t}+{U}_{f}\times {h}_{t-1}+{b}_{f})\end{eqnarray*}

(6) \begin{eqnarray*}{i}_{t}=\sigma ({W}_{i}\times {x}_{t}+{U}_{i}\times {h}_{t-1}+{b}_{i})\end{eqnarray*}

(7) \begin{eqnarray*}{o}_{t}=\sigma ({W}_{o}\times {x}_{t}+{U}_{o}\times {h}_{t-1}+{b}_{o})\end{eqnarray*}

(8) \begin{eqnarray*}{C}_{t}^{{^{\prime}}}=\tanh \nolimits ({W}_{c}\times {x}_{t}+{U}_{c}\times {h}_{t-1}+{b}_{c})\end{eqnarray*}

(9) \begin{eqnarray*}{C}_{t}=\sigma ({f}_{t}\odot {C}_{t-1}+{i}_{t}\odot {C}_{t}^{{^{\prime}}})\end{eqnarray*}

(10) \begin{eqnarray*}{h}_{t}=\tanh \nolimits ({C}_{t})\odot {o}_{t}\end{eqnarray*}

The processed data from the autoencoder is provided to the LSTM network. LSTM structure is shown above in Table 4.

**Table 4:** LSTM structure.

Layer (type)	Output shape	Param #
lstm_5 (LSTM)	(None, 2, 100)	42000
lstm_6 (LSTM)	(None, 256)	365568
dropout_4 (Dropout)	(None, 256)	0
dense_3 (Dense)	(None, 4)	1028
Total params: 408,596 Trainable params: 408,596 Non-trainable params: 0		

LSTM helps in extracting features from data using its multi-layered architecture. The architecture has two connected layers, and in the output layer, we have used the sigmoid activation function. In between the second layer of LSTM and fully connected layers, we have a dropout rate of 0.2. The dataset is iterated over 100 epochs with a batch size of 32. The model is further analyzed for performance metrics described in the literature review.

### GRU model

**Figure 4:**
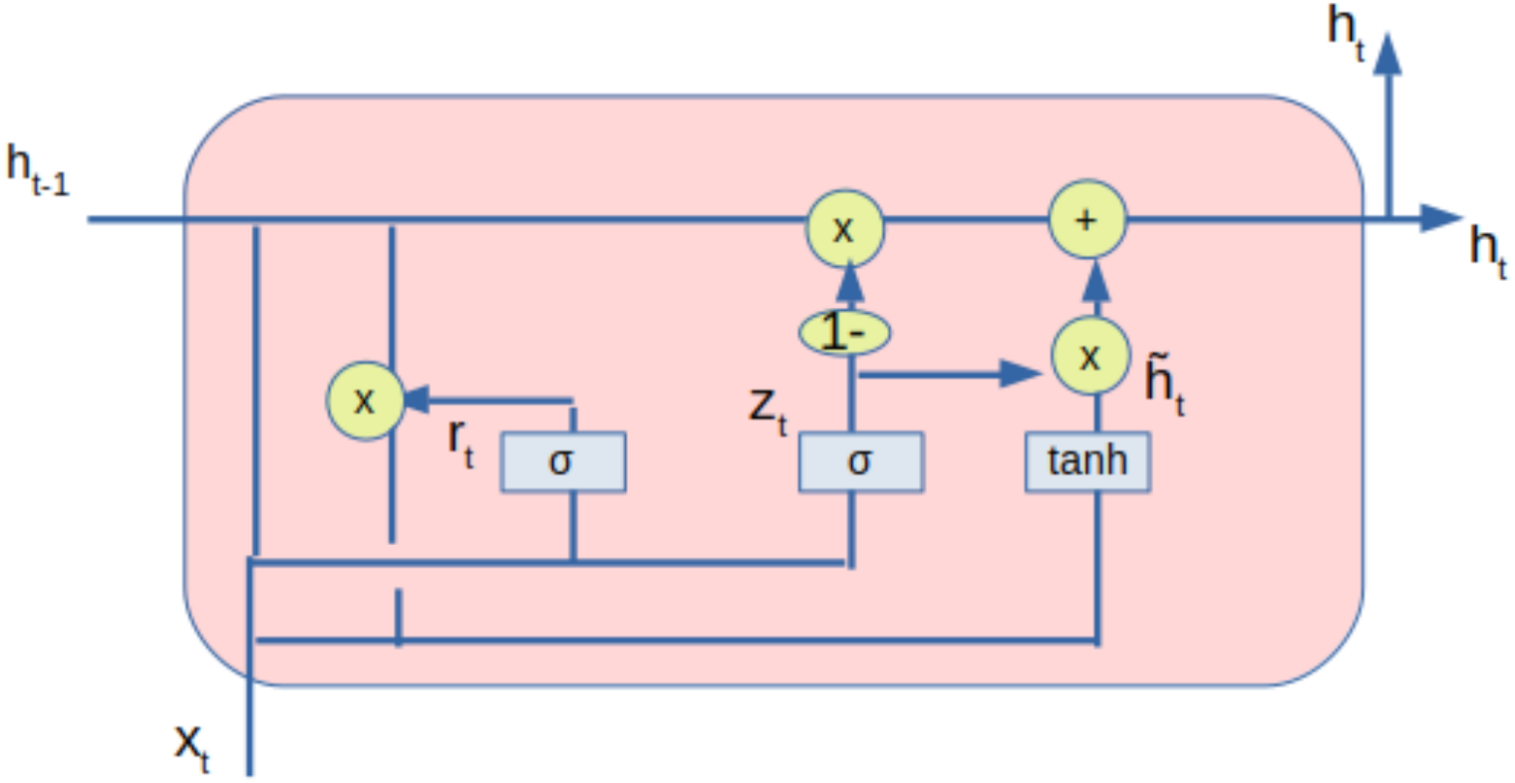
GRU with inputs and outputs.

The processed data from the autoencoder is also fed to the gated recurrent unit (GRU) network. GRU is also a type of RNN used in deep learning models. The architecture of the GRU is shown in Fig. 4. The GRU comprises of update gate (*z*_*t*_), reset gate (*r*_*t*_) and a current memory content ($\hat {{h}_{t}}$).The output of the GRU shown as (*h*_*t*_) is stored in the final memory. The update gate controls the input (*x*_*t*_) and previous output (*h*_*t*−1_) to be transferred to the next cell, which is regulated by (*W*_*z*_). The reset gate has deployed the extent of information to the forget gate. Only the relevant information is transferred to the next iteration based on the weight W.

The main operations by the GRU are given by the following equations:

(11) \begin{eqnarray*}{z}_{t}=\sigma ({W}_{z}\odot [{h}_{t-1},{x}_{t}])\end{eqnarray*}

(12) \begin{eqnarray*}{r}_{t}=\sigma ({W}_{z}\odot [{h}_{t-1},{x}_{t}])\end{eqnarray*}

(13) \begin{eqnarray*}\tilde {{h}_{t}}=tanh(W[{r}_{t}\times {h}_{t-1},{x}_{t}])\end{eqnarray*}

(14) \begin{eqnarray*}{h}_{t}=(1-{z}_{t})\times {h}_{t-1}+{z}_{t}\times {h}_{t}\end{eqnarray*}

where (*z*_*t*_) and (*r*_*t*_) are intermediate values available from the update and reset gates and tanh is the hyperbolic tangent function and *σ* is the sigmoid function.

The notations and descriptions used in GRU is given in Table 5. The Eqs. (11) and (12) represents the values of the update gate and reset gate respectively. The current state of the memory cells at time *t* are given by the Eqs. (13) and (14) represents the output state.

**Table 5:** GRU notations and descriptions.

**Notation**	**Description**
*z*_*t*_	Update gate
*r*_*t*_	Reset gate
*o*_*t*_	Output gate
$\tilde {{h}_{t}}$	Current memory content
*h*_*t*_	Output state
*h*_*t*−1_	Previous output state
*σ*	sigmoid
tanh	tanh
*W*, *W*_*z*_	Weights
×	Multiplication
⊙	Element wise multiplication

The structure of the GRU is shown in Table 6. The model hyperparameters in GRU are optimized using Grid Search.

**Table 6:** GRU structure.

Layer (type)	Output shape	Param #
gru_5 (GRU)	(None, 2, 100)	31800
gru_2 (GRU)	(None, 256)	274176
dropout_1 (Dropout)	(None, 256)	0
dense_1 (Dense)	(None, 5)	1285
Total params: 307, 261 Trainable params: 307, 261 Non-trainable params: 0		

**Table 7:** Summary of results of evaluation metrics without deep autoencoder.

Evaluation metrics	LSTM	LSTMSA	GRU	GRUSA
RMSE	1.09	1.08	1.05	1.01
MAE	0.7638	0.7401	0.7285	0.6894
MAPE	1.6356	1.3824	1.4794	1.2502
R2	0.9768	0.9771	0.9803	0.9830

GRU overcomes the disadvantages of RNN and also eliminates the vanishing gradient problem. It helps in extracting features from data using its multi-layered architecture.

The architecture has two connected layers, and we use an update gate and a reset gate here. We have used the sigmoid function in the output layer. In between the second layer of GRU and fully connected layers, we have accommodated a dropout rate of 0.2. The dataset is iterated over 100 epochs with a batch size of 32. The model is further analyzed for performance metrics described in the literature review.

### VADER

**Figure 5:**
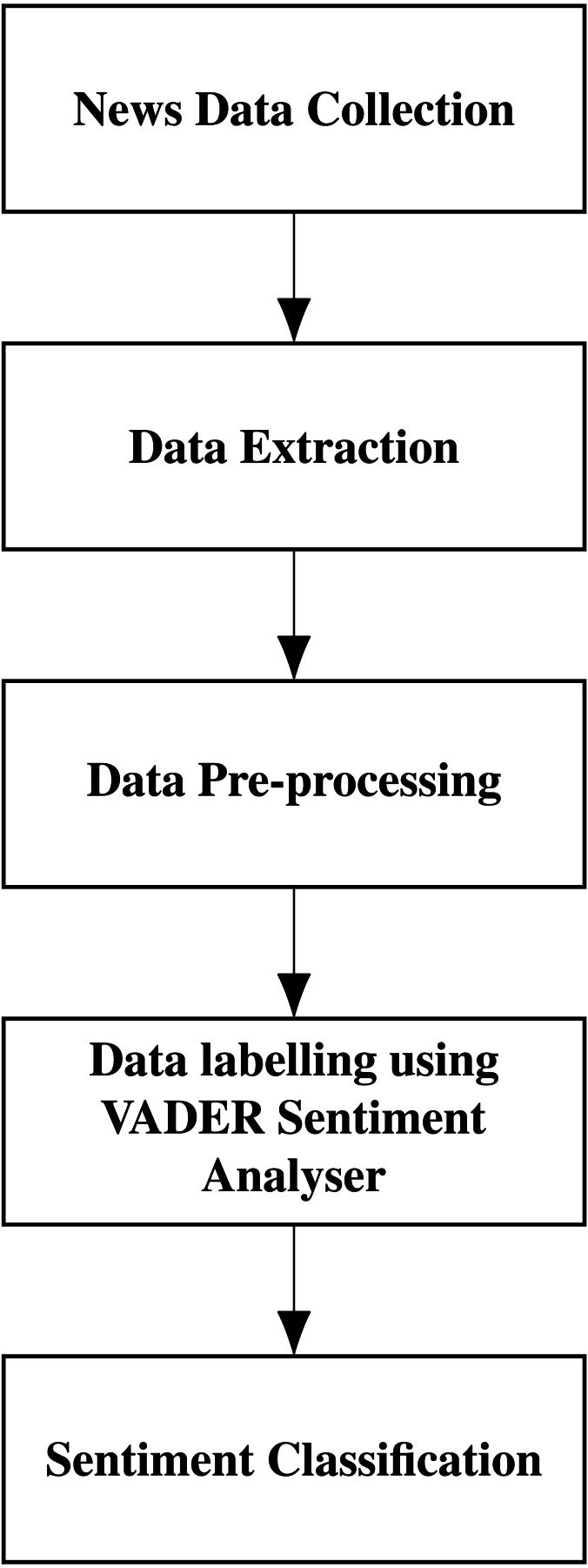
Sentiment classification of news headlines.

The sentiment analysis is performed using VADER (Valence Aware Dictionary for sEntiment Reasoning) and creates a score index. Introduced by Hutto & Gilbert (2014), VADER is an integrated version of rule-based and lexicon-based software. It can detect the polarities and the sentiment intensity in the news. Researchers commonly use VADER as it is open-source, free to use, and good in speed performance tradeoffs. This software uses fewer resources than other machine learning-based models and needs a lesser amount of training data. VADER is part of the natural language processing package NLTK. We have analyzed the performance of VADER and Textblob and got better results for VADER. The sequence of steps in sentiment classification is shown in Fig. 5.

 To perform sentiment analysis using VADER, we read the news headlines and pre-processed the news headlines by cleaning them. We calculate the polarity compound, positive, negative, and neutral using the SentimentIntensityAnalyzer. The polarity score is taken, and the sentiment is classified as positive or negative. The positive news is given a value of 1, and the negative news is given a value of 0.

The experimental results are discussed in the next section.

## Results and Discussions

In order to check the performance of the proposed model, we have considered the stock of a reputed company and validated the results in this section. We have also compared the result with other state-of-the-art methods in literature (Dey et al., 2021). The experiment was performed on an i5, 2.7 GHz processor with 8 GB RAM, and we used the Linux operating system. The computations are done in Python open source programming language. We have done work with DAE and LSTM using stock dataset, which is accepted for publication, and have obtained reasonable results with this model. This work is adopted in this model, and sentimental analysis is combined with it to enhance the performance of the model. The experimental analysis is performed with our accepted DAE-LSTM model for the stock dataset we considered here for our study. The results show that the DAE-LSTM model works better than considering the LSTM model alone. The final results show that passing historical data through DAE and combining it with LSTM/GRU, and concatenating with sentimental score gives better results than the stand-alone models.

### Dataset

The experimental dataset is divided into two parts: the historical dataset and news headlines to obtain the sentiment index. We consider the period from 01/03/2000 to 01/07/2016 for daily stock prices and news articles of the company in this study. The daily stock prices contain six values Open, High, Low, Close, Adjusted Close, and Volume. We are considering the Open, High, Low, and Close (OHLC) values for historical data in this is collected from Yahoo finance. The top 25 news on a particular day about the stock was considered for the study. The news dataset used in this study for sentiment analysis with news headlines was extracted from the Kaggle community. We conducted experiments to evaluate the performance of the proposed method. To compare the performance of the model with state-of-the-art methods, we have taken three types of datasets, namely Honda Motor Company (HMC), Oracle Corporation (ORCL), and Intuit Inc. (INTU) data. The historical data was collected from Yahoo finance and evaluated under the same conditions. The detailed analysis and results are given in the following sections.

### Training and validation loss

A graph of the learning performance of the model with time is given by the learning curve. In machine learning, especially in deep learning neural networks, we can use learning curves as a diagnostic tool for problems that learn from training datasets incrementally. From the learning curve, we can evaluate the performance of the model on train and validation sets to know whether the model is underfit, overfit, or goodfit. The *x*-axis indicates the time or epochs, whereas the *y*-axis denotes the line plot of learning. The training loss shows how accurately the model fits the training data, and the validation loss shows how well the model fits new data. Loss measures the goodness of the model and the smaller the loss, the better the relationship of the input data and output and hence better our model. Training loss is measured during each epoch, while validation loss is measured after each epoch. If there is a minimal gap between the training loss and validation loss that decreases to the point of stability, we can say that our model is a good fit.

**Figure 6:**
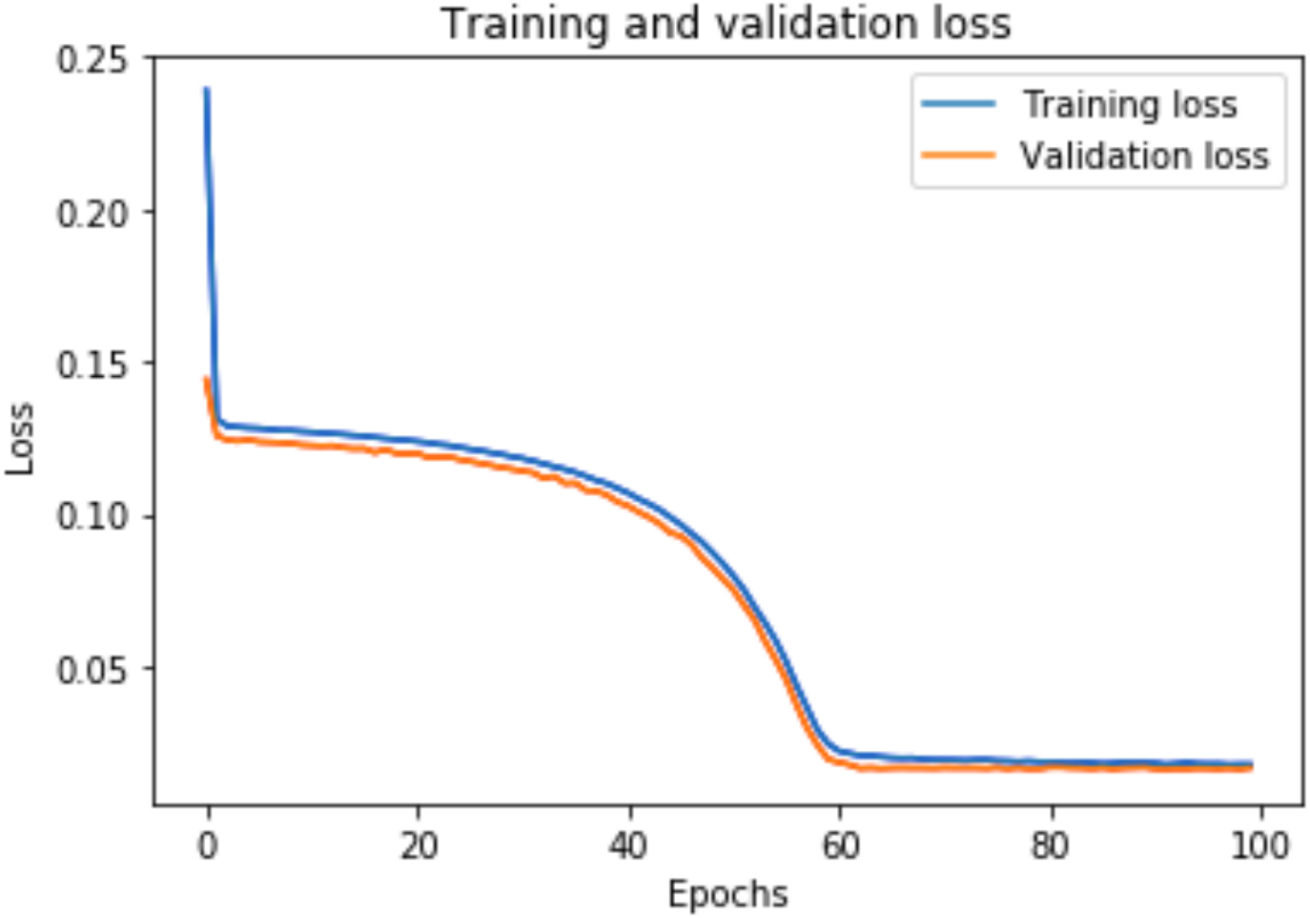
Training and validation loss for DAE-LSTM model.

**Figure 7:**
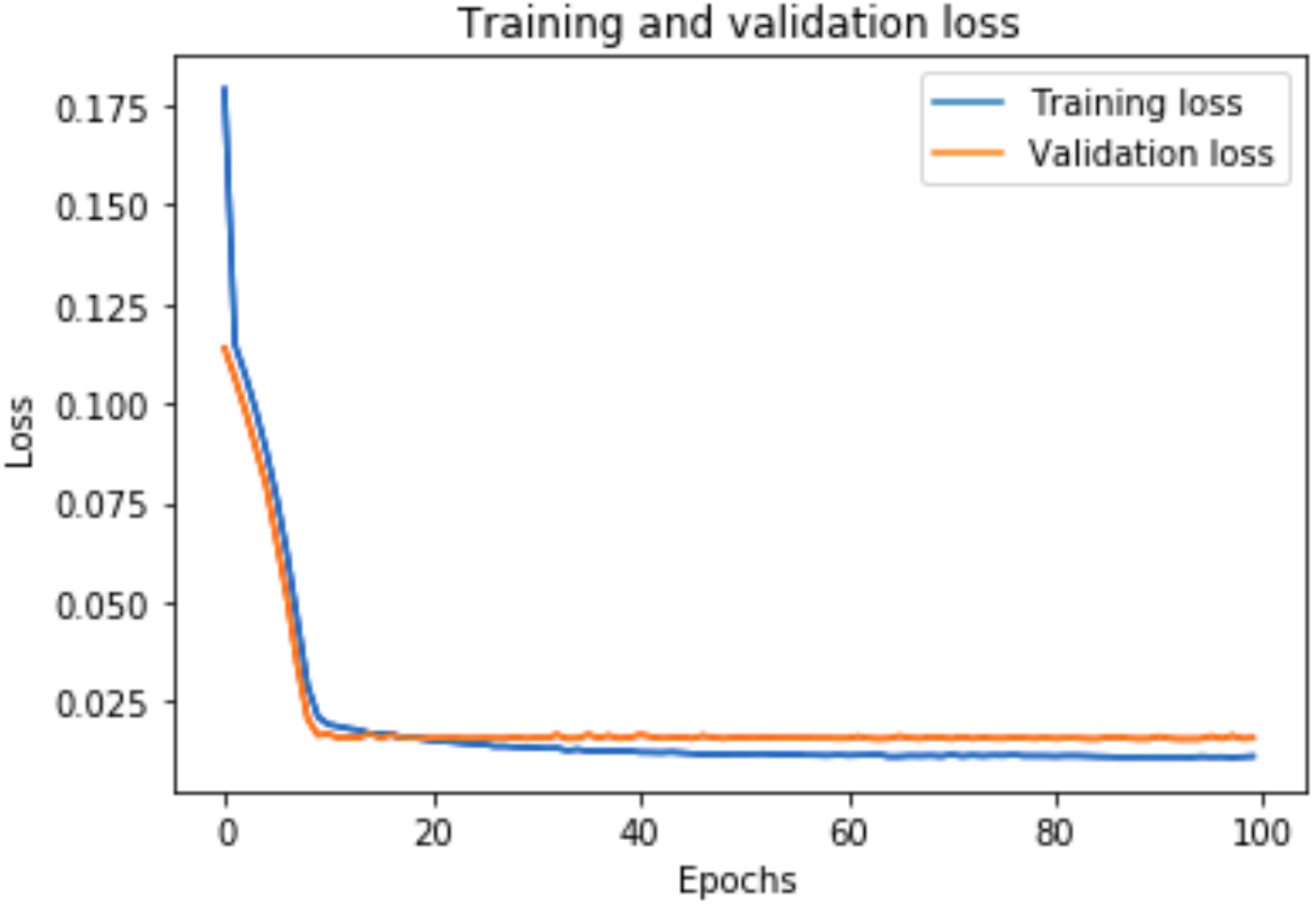
Training and validation loss for DAE-GRU model.

The training loss and validation loss are obtained after training the model for 100 epochs. Figure 6 shows the training and validation loss for the DAE-LSTM model, and Fig. 7 shows the training and validation loss for the DAE-GRU model. From the graphs, we can infer a minimal gap between the two final loss values, which shows that the model is a good fit.

**Figure 8:**
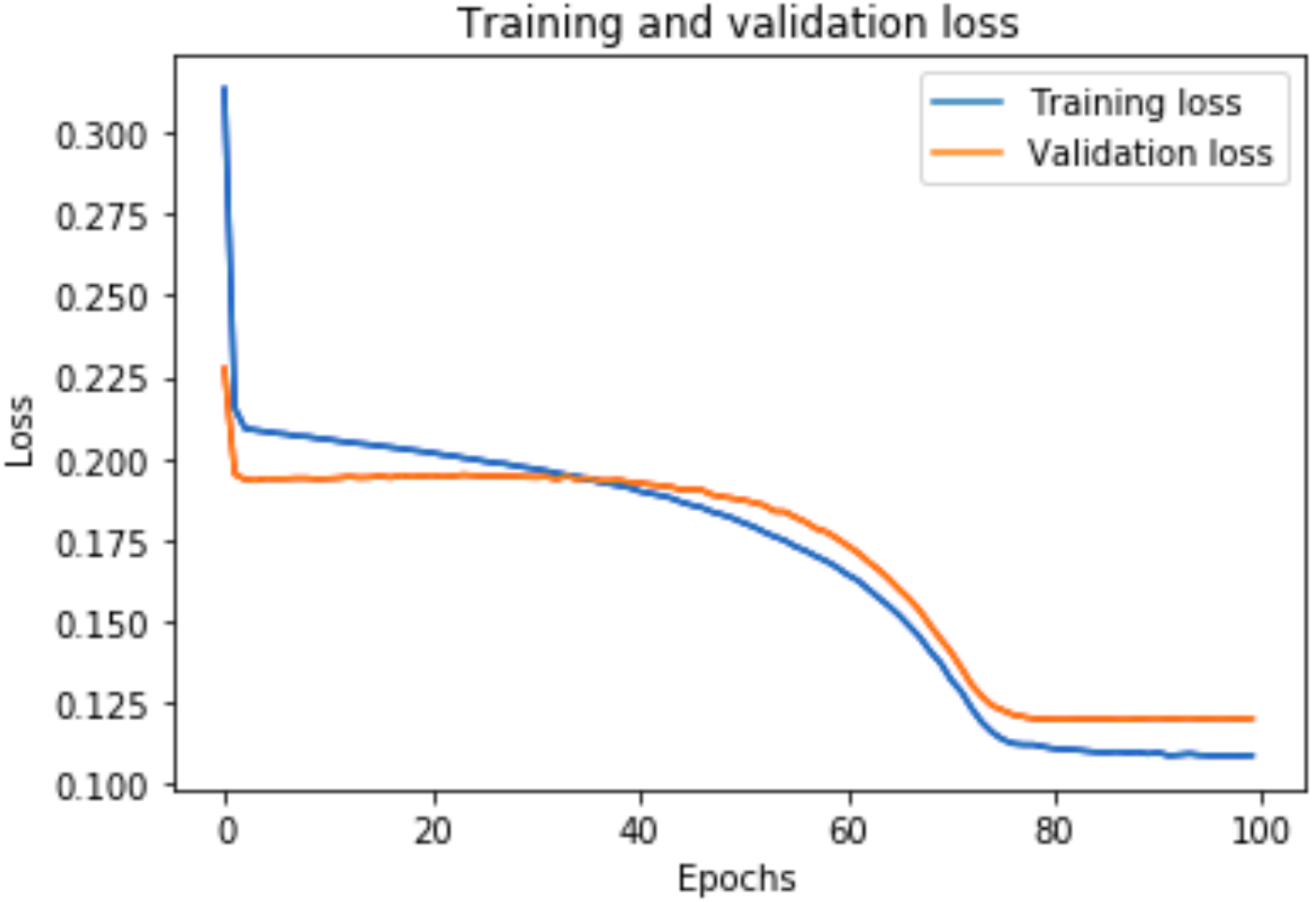
Training and validation loss for DAE-LSTMSA.

**Figure 9:**
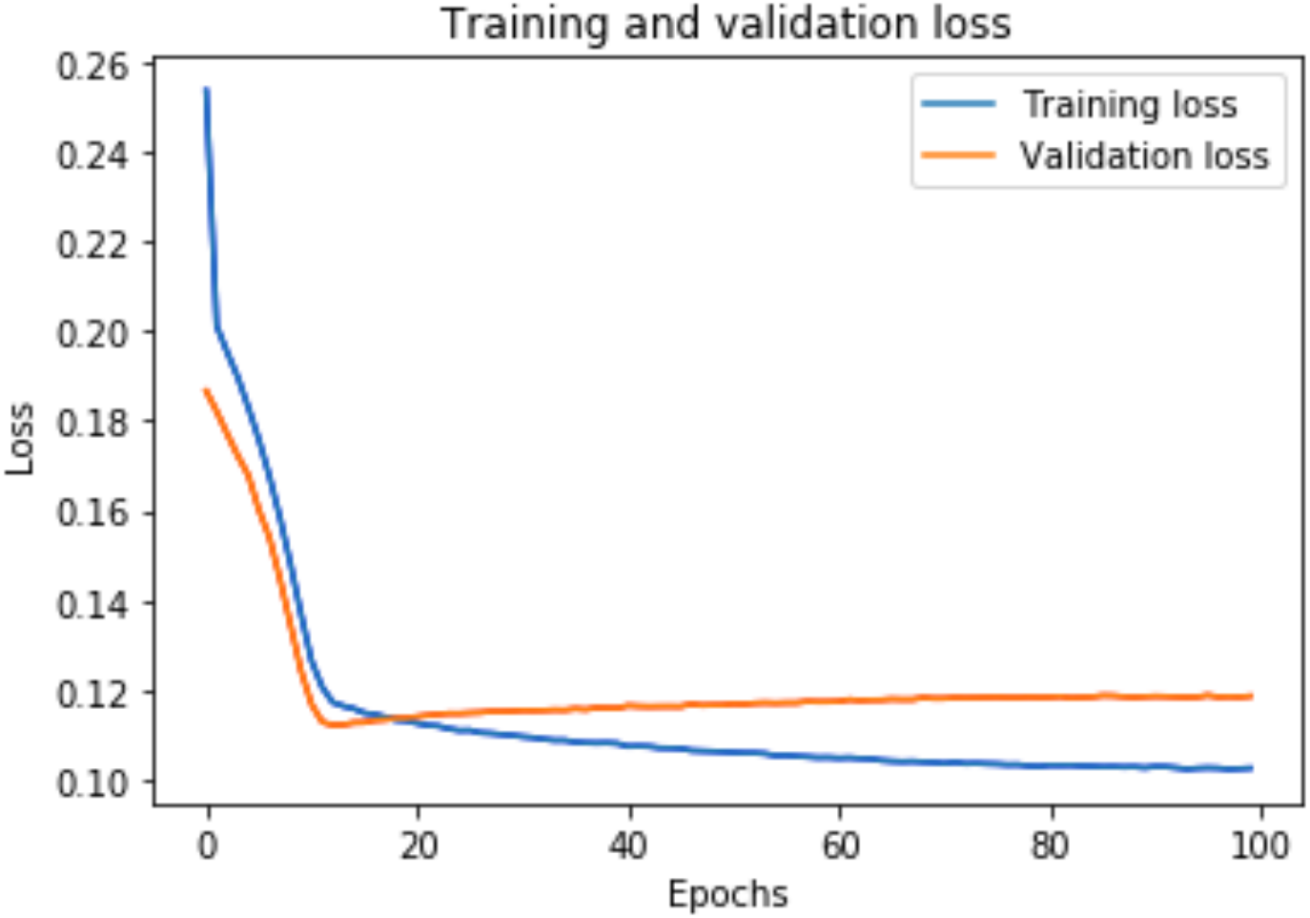
Training and validation loss for DAE-GRUSA.

Figure 8 shows the training and validation loss for the DAE-LSTMSA model, and Fig. 9 shows the training and validation loss for the DAE-GRUSA model. It is evident from the graphs that the loss is converging, which indicates that the model is a good fit.

### Evaluation metrics

We used different performance metrics described in the literature to assess the correctness of the stock price prediction (Ding & Qin, 2020). The metrics used in our research are the root mean square error (RMSE), mean absolute error (MAE), mean absolute percentage error (MAPE), and R-squared (*R*^2^) to measure the goodness of fit between the actual and predicted values. The metrics can be expressed as in Eqs. (15), (16), (17) and (18).

(15) \begin{eqnarray*}\text{RMSE}=\sqrt{ \frac{1}{n} \sum _{i=1}^{n}({y}_{i}-{\hat {y}}_{i})^{2}}\end{eqnarray*}

(16) \begin{eqnarray*}\text{MAE}= \frac{1}{n} \sum _{i=1}^{n} \left\vert ({y}_{i}-{\hat {y}}_{i}) \right\vert \end{eqnarray*}

(17) \begin{eqnarray*}\text{MAPE}= \frac{100\text{%}}{n} \sum _{i=1}^{n} \left\vert \frac{({y}_{i}-{\hat {y}}_{i})}{{y}_{i}} \right\vert \end{eqnarray*}

(18) \begin{eqnarray*}{R}^{2}=1- \frac{(\sum _{i=1}^{n}({y}_{i}-{\hat {y}}_{i})^{2})/n}{(\sum _{i=1}^{n}(\bar {{y}_{i}}-{\hat {y}}_{i})^{2})/n} .\end{eqnarray*}

**Figure 10:**
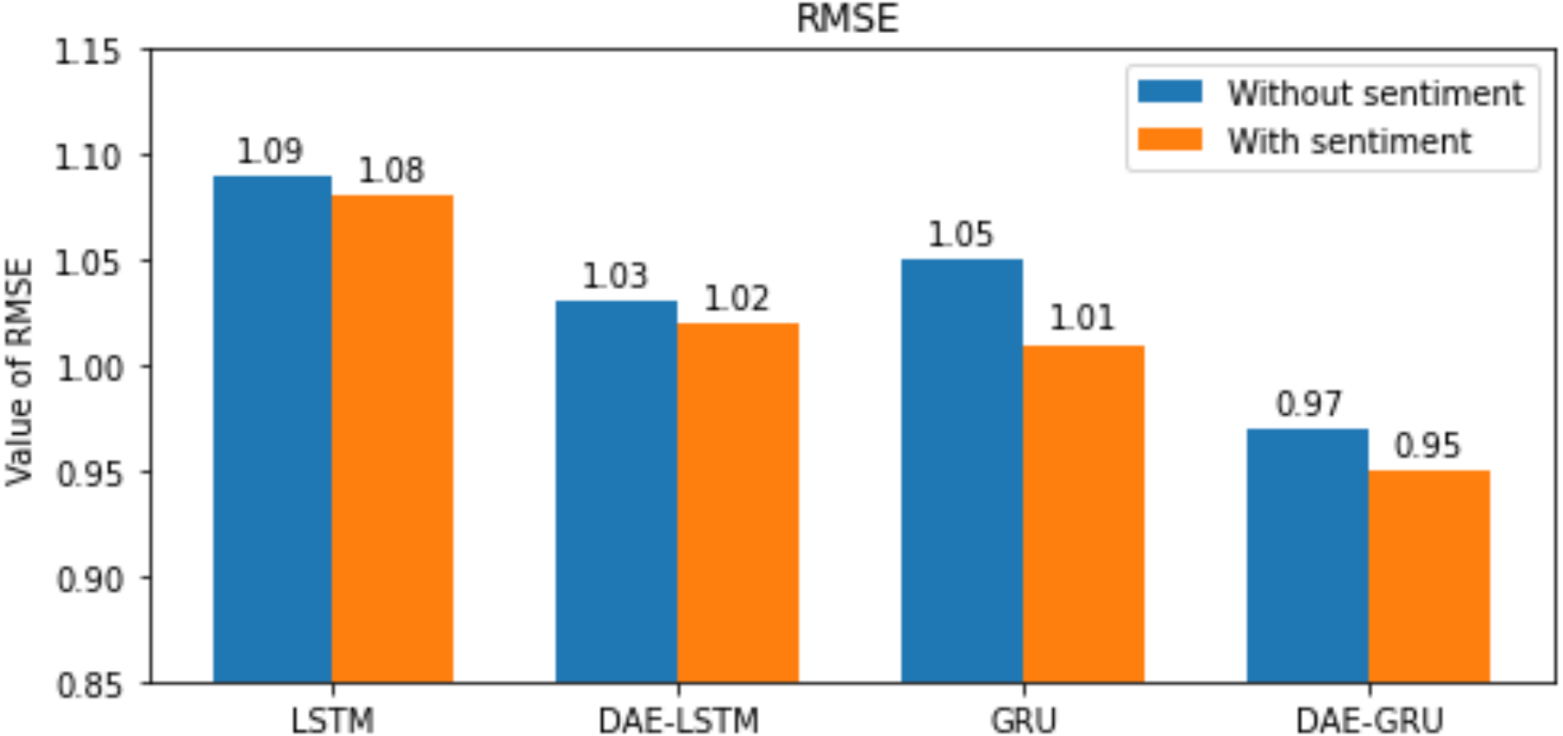
RMSE for each model.

**Figure 11:**
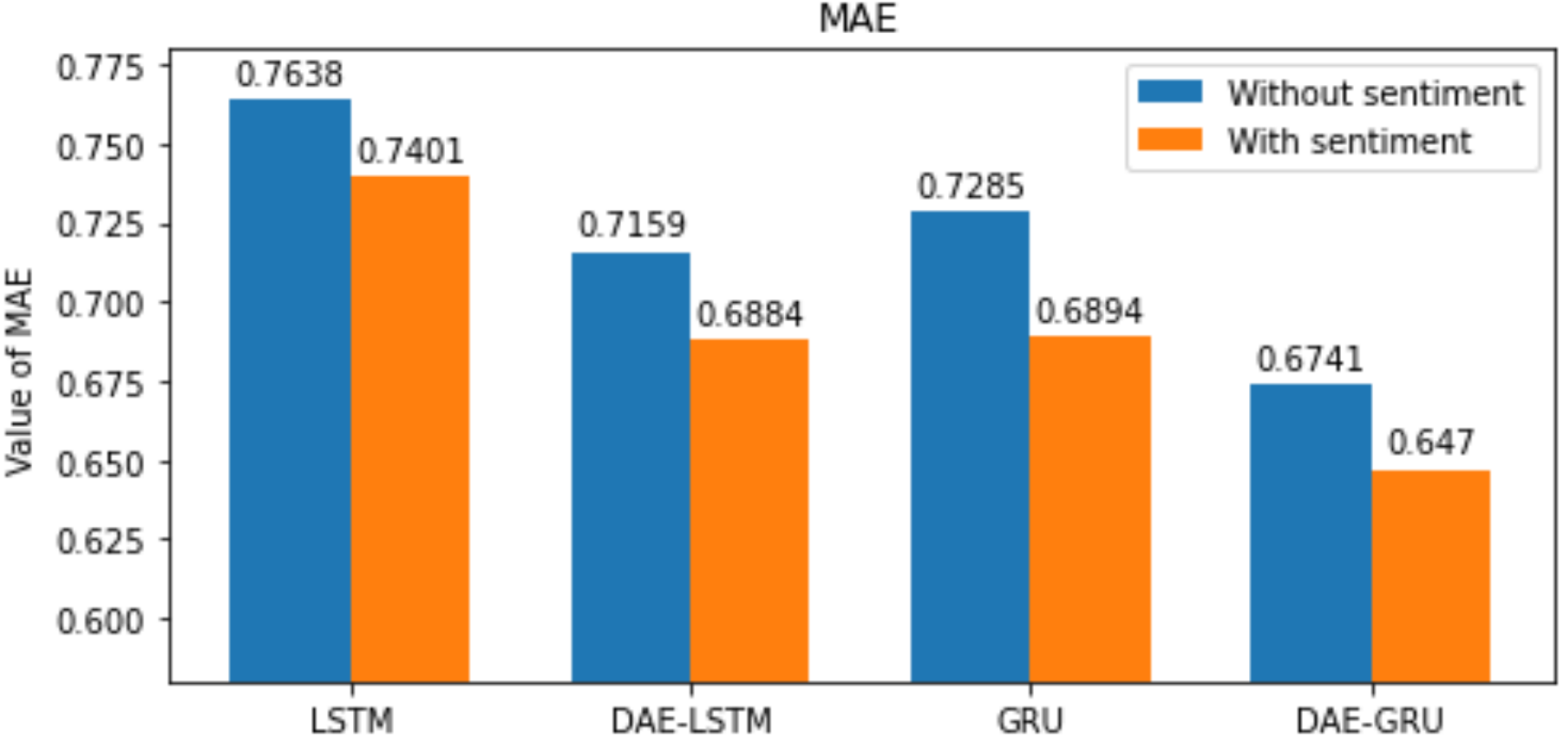
MAE for each model.

**Figure 12:**
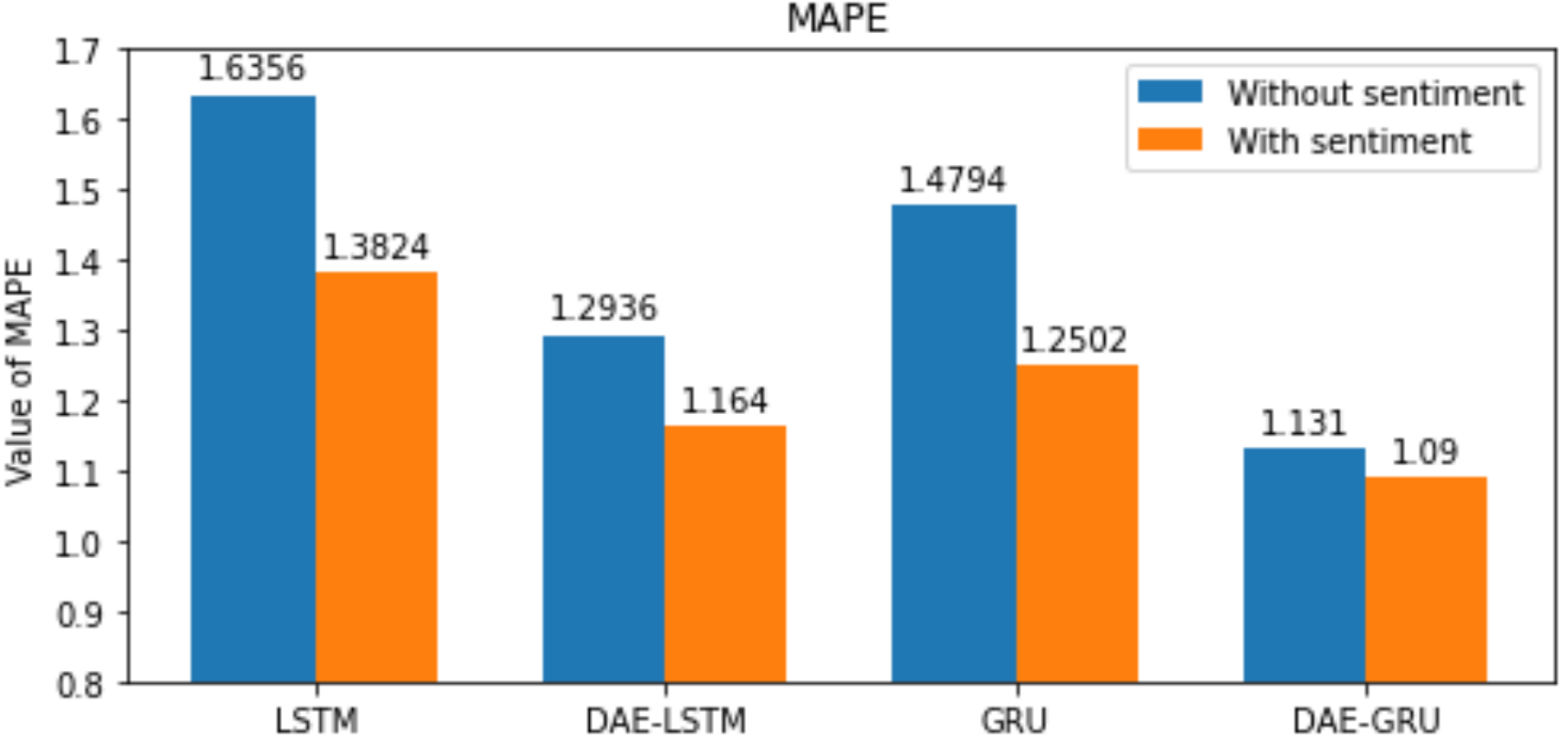
MAPE for each model.

**Figure 13:**
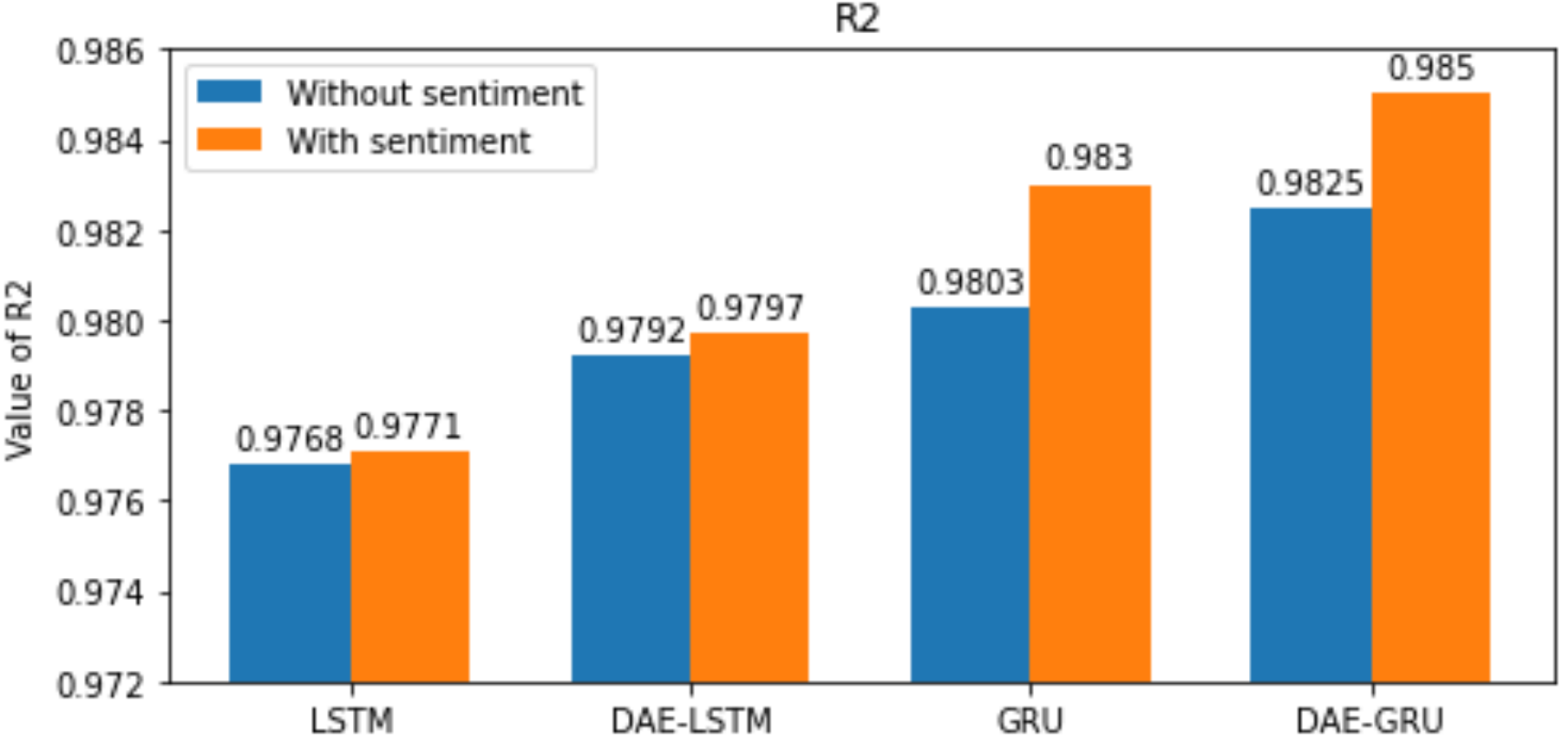
R-squared for each model.

Here n is the total number of samples, *y*_*i*_ and ${\hat {y}}_{i}$, indicates the actual value and predicted value of the test set; and the mean of real values of the test set is represented by $\bar {{y}_{i}}$. The lower the value of RMSE, MAE, and MAPE better the model. The range of *R*^2^ is normally between 0 and 1. The closer the value of *R*^2^ to 1 better the model’s ability to fit the data. The evaluation metrics are represented in the bar graphs in Figs. 10, 11, 12 and 13.

### Performance comparison

In this section, we compare the LSTM and GRU models with and without deep autoencoder. To make the cooperative model consistent in this research, both LSTM and GRU have the same number of layers. The models are executed with an epoch value of 100 and batch size of 32 to evaluate the performance. Two input layers, followed by an LSTM/GRU layer, succeeded by a dropout layer, and finally, a dense output layer, constitutes the model structure. The hyperbolic tangent is the activation function used in each layer of LSTM/GRU. We are dividing the total data set comprising 4,101 samples into 2,748 training samples and 1,353 test samples. The two input sets used in the experiment are historic stock values and the news sentiment labels. We used these inputs to train the LSTM/GRU models separately.

Our study aims to measure the LSTM and GRU model’s performance under different conditions, as shown in Tables 7 and 8. After passing the historical dataset through a deep autoencoder, it is observed that the GRU model with news sentiments performs better than the LSTM counterpart under the same cooperative environment. We can see that news sentiment has an influential impact on stock market prediction.

**Figure 14:**
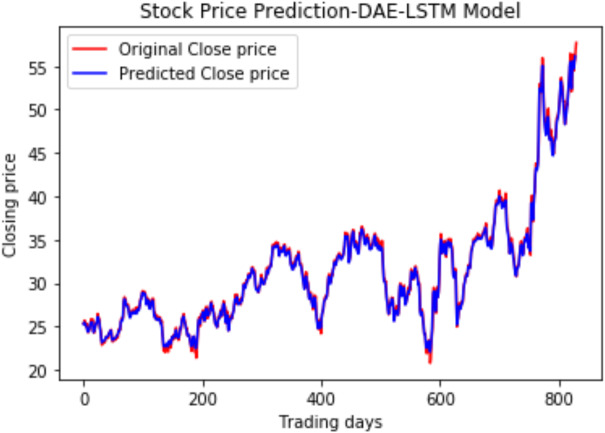
Stock close price prediction DAE-LSTM model.

**Figure 15:**
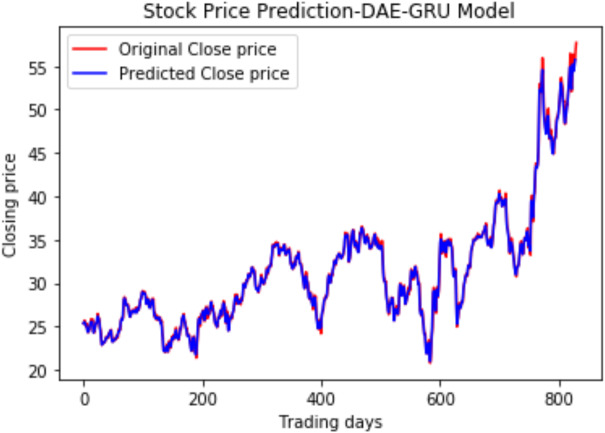
Stock close price prediction DAE-GRU model.

We have plotted the trading days on the *X*-axis and the close price on the *Y*-axis. The original test close price and predicted test close price are shown in the plots. By observing the test plots of the two models in Figs. 14 and 15, it is evident that GRU performs better than LSTM in the models DAE-LSTM and DAE-GRU. The lower the value of the evaluation metrics RMSE, MAPE, and MAE better is the model. An R2 value close to 1 indicates a better model.

**Figure 16:**
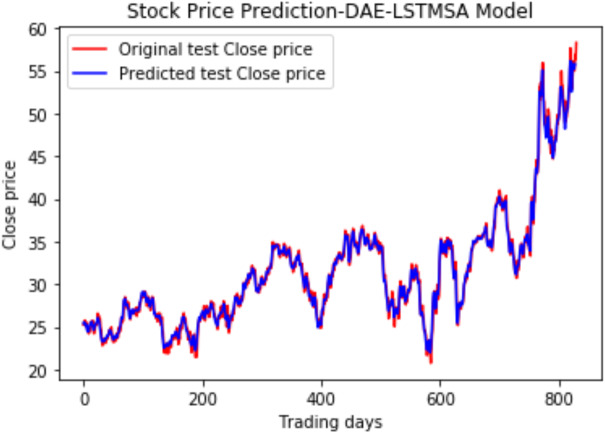
Stock close price prediction DAE-LSTMSA model.

**Figure 17:**
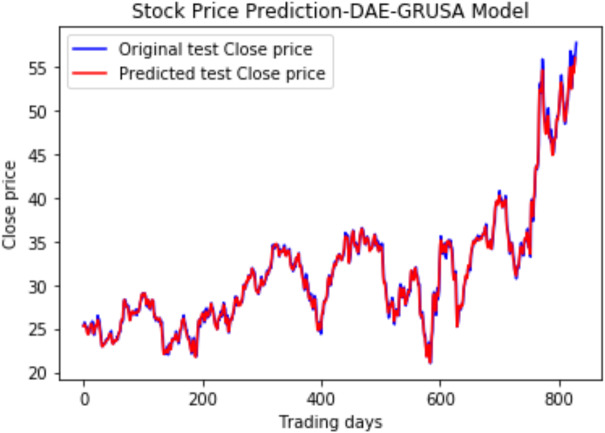
Stock close price prediction DAE-GRUSA model.

Under the same cooperative environment, the DAE-LSTMSA model performs better than the DAE-LSTM model, and the DAE-GRUSA model performs better than the DAE-GRU model. The performance is improved by using sentiment analysis with news headlines and historical stock datasets than considering historical stock prices alone while using the GRU model. DAE-LSTMSA and DAE-GRUSA models perform better than the DAE-LSTM and DAE-GRU models in performance metrics RMSE, MAPE, MAE, and R2. The test plots of Figs. 16 and 17 have a trading price on the *X*-axis and a close price on the *Y*-axis. For the models DAE-LSTMSA and DAE-GRUSA, we have plotted the original test close price and predicted test close price. From the test plots of the two models in Figs. 16 and 17 and the data shown in the tables, it is clear that the DAE-GRUSA model provides better results than all the other models.

**Table 8:** Summary of results of evaluation metrics with deep autoencoder.

Evaluation metrics	DAE-LSTM	DAE-LSTMSA	DAE-GRU	DAE-GRUSA
RMSE	1.03	1.02	0.97	0.95
MAE	0.7159	0.6884	0.6741	0.6470
MAPE	1.2936	1.1640	1.1310	1.09
R2	0.9792	0.9797	0.9825	0.9850

There are relatively few studies in the literature indicating the relationship between the stock price and news headlines. Our model introduces a new method by incorporating a deep autoencoder to denoise the historical stock data and then combine it with labeled sentiments. The results reveal that using news headlines significantly improves the performance of stock price prediction. The results indicate that deep learning models integrated with autoencoder and sentiment analysis are better equipped to predict stock prices. Financial markets are heavily affected by other market sentiments such as twitter data and other external factors, which are not considered in this algorithm which is a shortcoming of this model.

### Comparison with state-of-the-art models

We have also compared the performance of our model with similar models in recent literature given in Dey et al. (2021) using the same data sets. The performance comparison is done based on three different stock data sets of HMC, ORCL, and INTU. The comparison is shown in Tables 9, 10 and 11. It is observed that the proposed models perform better than the benchmark LSTM and GRU models.

**Table 9:** Comparison with state-of-the-art models: HMC stock.

Evaluation metrics	LSTM (State of the art)	DAE-LSTM (Proposed model)	GRU (State of the art)	DAE-GRU (Proposed model)
RMSE	0.7970	0.52	0.7811	0.37
MAE	0.5807	0.407	0.5695	0.2696
MAPE	2.1027	1.43	2.1001	0.9601
R2	0.9409	0.97	0.9606	0.9854

**Table 10:** Comparison with state-of-the-art models: ORCL stock.

Evaluation metrics	LSTM (State of the art)	DAE-LSTM (Proposed model)	GRU (State of the art)	DAE-GRU (Proposed model)
RMSE	1.3969	1.13	1.4077	1.02
MAE	0.9862	0.9599	0.9996	0.8156
MAPE	1.9787	1.9605	2.0192	1.6297
R2	0.9015	0.9514	0.90	0.9604

**Table 11:** Comparison with state-of-the-art models: INTU stock.

Evaluation metrics	LSTM (State of the art)	DAE-LSTM (Proposed model)	GRU (State of the art)	DAE-GRU (Proposed model)
RMSE	10.55	10.44	7.6649	7.46
MAE	7.8352	6.4967	4.9903	3.90
MAPE	3.4130	2.1205	2.3146	1.9945
R2	0.9664	0.9849	0.9822	0.9875

## Conclusion and Future Scope

A cooperative deep-learning architecture for stock market prediction using the deep autoencoder, LSTM, GRU, and sentiment analysis with news headlines was proposed in this work. The performance of our DAE-LSTMSA and DAE-GRUSA models was evaluated under the same conditions and compared with the benchmark DAE-LSTM and DAE-GRU models. Both models show lower error rates indicating better performance while using news headlines and the historical stock dataset. Results indicate that our models perform better than the state-of-the-art models in the literature.

We demonstrated that the DAE-GRUSA model provides better results than other models. We conclude that sentiment analysis with news significantly influences stock market prediction. This work is significant to the community because prediction problems involving dynamic stock data are challenging and have yet to reach maturity. Our proposed model performs reasonably well, but there is further scope for improvement by incorporating sentiment analysis using Twitter data and historical datasets. As a future scope, we can include Twitter data instead of using news sentiments alone. In addition, an advanced hyper-parameter selection may be used to optimize the proposed deep learning model. There is also scope for attempting the model on time series problems in other domains involving sequential data and sentiments.

## Additional Information and Declarations

### Data Availability

The following information was supplied regarding data availability: The data is available at Zenodo: Rekha KS. (2022). A cooperative deep learning model for stock market prediction using deep autoencoder and sentiment analysis (Version 1) [Data set]. Zenodo. https://doi.org/10.5281/zenodo.7044026.
